# Evaluating diagnostic tests for bovine tuberculosis in the southern part of Germany: A latent class analysis

**DOI:** 10.1371/journal.pone.0179847

**Published:** 2017-06-22

**Authors:** Valerie-Beau Pucken, Gabriela Knubben-Schweizer, Dörte Döpfer, Andreas Groll, Angela Hafner-Marx, Stefan Hörmansdorfer, Carola Sauter-Louis, Reinhard K. Straubinger, Pia Zimmermann, Sonja Hartnack

**Affiliations:** 1 Clinic for Ruminants with Ambulatory and Herd Health Services, Centre for Clinical Veterinary Medicine, LMU Munich, Oberschleißheim, Germany; 2 Food Animal Production Medicine, School of Veterinary Medicine, University of Wisconsin, Madison, Wisconsin, United States of America; 3 Workgroup Financial Mathematics, Department of Mathematics, LMU Munich, Munich, Germany; 4 Bavarian Health and Food Safety Authority, Oberschleißheim, Germany; 5 Institute for Epidemiology, Friedrich-Loeffler-Institute, Greifswald, Germany; 6 Institute for Infectious Diseases and Zoonoses, Department of Veterinary Science, LMU Munich, Munich, Germany; 7 Section of Epidemiology, Vetsuisse Faculty, University of Zurich, Zurich, Switzerland; Universidade Federal de Pelotas, BRAZIL

## Abstract

Germany has been officially free of bovine tuberculosis since 1996. However, in the last years there has been an increase of bovine tuberculosis cases, particularly in the southern part of Germany, in the Allgäu region. As a consequence a one-time tuberculosis surveillance program was revisited with different premortal and postmortal tests. The aim of this paper was to estimate diagnostic sensitivities and specificities of the different tests used within this surveillance program. In the absence of a perfect test with 100% sensitivity and 100% specificity, thus in the absence of a gold standard, a Bayesian latent class approach with two different datasets was performed. The first dataset included 389 animals, tested with single intra-dermal comparative cervical tuberculin (SICCT) test, PCR and pathology; the second dataset contained 175 animals, tested with single intra-dermal cervical tuberculin (SICT) test, Bovigam® assay, pathology and culture. Two-way conditional dependencies were considered within the models. Additionally, inter-laboratory agreement (five officially approved laboratories) of the Bovigam® assay was assessed with Cohen's kappa test (21 blood samples). The results are given in posterior means and 95% credibility intervals. The specificities of the SICT test, SICCT test, PCR and pathology ranged between 75.8% [68.8–82.2%] and 99.0% [96.8–100%]. The Bovigam® assay stood out with a very low specificity (6.9% [3.6–11.1%]), though it had the highest sensitivity (95.7% [91.3–99.2%]). The sensitivities of the SICCT test, PCR, SICT test, pathology and culture varied from 57.8% [48.0–67.6%] to 88.9% [65.5–99.7%]. The prevalences were 19.8% [14.6–26.5%] (three-test dataset) and 7.7% [4.2–12.3%] (four-test dataset). Among all pairwise comparisons the highest agreement was 0.62 [0.15–1]). In conclusion, the specificity of the Bovigam® assay and the inter-laboratory agreement were lower than expected.

## Introduction

Bovine tuberculosis (bTB) which is caused by *Mycobacterium caprae* and *Mycobacterium bovis* is an important public and animal health problem and an international trade issue in Europe and worldwide [1–6]. Therefore, using reliable, fast and cost-effective diagnostic methods is essential for the control of bTB.

National control programs rely on testing of cattle and removal of animals which are diagnosed as positive [7]. Infected animals are detected with tuberculin skin tests or the Bovigam® gamma-interferon (IFN-ɣ) assay. The tuberculin skin test, the prescribed test for international trade, is used as a single intradermal cervical tuberculin (SICT) test or single intra-dermal comparative cervical tuberculin (SICCT) test in Europe and as caudal fold tuberculin (CFT) test in North America, Australia and New Zealand [7, 8]. Accuracy of the skin tests varies widely due to different factors, affecting the host and the test itself. The exact estimation of the test characteristics in the field is therefore difficult [8–10]. However, the intradermal skin tests normally have a high specificity although sensitivity can be somewhat lower [9]. The low test sensitivity and the logistics of holding cattle for 3 days to read the test has led to the development of the Bovigam® assay, in 1985 [11]. Australia was the first country to officially accept this test for the diagnosis of bovine tuberculosis in 1991 [11]. In comparison to the skin test the Bovigam® assay almost always showed a better sensitivity but an equal or inferior specificity. The Bovigam® assay is supposed to have the ability to detect bTB earlier in the course of infection [11, 12]. In many countries it is used for serial or parallel testing together with the intradermal skin tests [7, 13]. For post-mortem diagnosis of previously positive-tested animals, bacteriological culture and PCR can be used following necropsy [1, 14, 15].

In many countries the test-and-cull regime led to the status Officially Bovine Tuberculosis free (OTF) [9, 16, 17]. Germany reached this status in 1997 [18]. Owing to the OTF status nationwide periodic surveillance using intradermal skin testing was replaced with surveillance by official meat inspection at the abattoir [19, 20]. Remarkably, there is an apparent increase of bTB cases since 2007, many of them detected during routine meat inspections and with a particular high prevalence in the southern part of Germany. These unexpected bTB cases led to a revision of the tuberculosis regulations in 2009, 2012 and 2013 with commencement of the act in 2009, 2013 and 2014. Within this revision the Bovigam® assay and the PCR were added as new diagnostic methods. Furthermore, the increase of bTB cases lead to the implementation of a one-time tuberculosis surveillance program in Germany in 2012 to verify the nation’s OTF status [21]. At the beginning of this surveillance program the SICCT test or a serial testing with the SICT test and the Bovigam® assay were used for ante-mortem diagnosis [22]. This was the first time that the Bovigam® assay was used as a field test in Germany. However, as a consequence of irregular test results, the testing regime was changed in March 2013 with the SICCT test as the only ante-mortem test. Moreover, the PCR analysis as described in the national *Official Collection of Methods* was used since then as additional post-mortem method [1, 22–24].

As described in the literature the sensitivities and specificities of bTB diagnostic tests vary widely [12, 13, 25, 26]. This leads to difficulties in identifying truly infected animals as well as in identifying risk factors for bTB [27]. Diagnostic accuracies of bTB diagnostic tests are often estimated using bacteriological culture as the so-called gold standard for confirmation of bTB [26, 28–30]. A gold standard is considered as a test that has known properties with a high sensitivity and specificity. Because bacteriological culture has limitations in sensitivity this may lead to a misclassification of data [13, 31]. By using a latent class approach the test characteristics can be assessed in the absence of a gold standard [32–34]. This latent class approach can be used within a Bayesian model and is based on multiple tests performed on the same animals. "Latent class" refers to the fact that the true disease state is always hidden [35]. In the *Standard Operating Procedure for OIE Registration of Diagnostic Kits* the Bayesian inference and latent class models are described to use for estimation of diagnostic sensitivities and specificities [36].

The aim of this study was to assess the diagnostic accuracies of the tests used within the bTB surveillance program in Germany between 2010 and 2014. To the best of our knowledge a latent class analysis for the diagnostic tests of bTB has never been applied in Germany.

## Material and methods

With the aim to obtain robust diagnostic test accuracy estimates for different pre- and post-mortem tests diagnosing bovine tuberculosis, a Bayesian latent class approach was performed. Regarding the Bovigam® assay agreement between blood samples tested by five different laboratories and between blood samples taken from two different anatomical locations was assessed with Cohen’s kappa coefficient.

### Ethics statement

The test results which were used for the Bayesian latent class approach were collected within the context of the officially ordered tuberculosis-surveillance program ("Untersuchungsprogramm: Rindertuberkulose in den Landkreisen der Alpenkette; AZ: 42a-G8755-2013/2-450) prior to this study and were not specifically taken for the purpose of this study. The program was conducted according to Directive 64/432/EEC on animal health problems affecting intra-EU trade in bovine animals and swine, Council Directive 80/219/EEC of 22 January 1980 amending Directive 64/432/EEC as regards tuberculosis and brucellosis and Council Directive 97/12/EC of 17 March 1997 amending and updating Directive 64/432/EEC on health problems affecting intra-Community trade in bovine animals and swine [37–39] to verify the OTF status.

The data used for the Bovigam® assay agreement existed prior to our research. The blood samples were taken in the context of the study "Optimierung der Methode Bovigam®—Test beim Rind—vergleichende Untersuchungen an 21 Tieren des Betriebs Spitalhof, Kempten" which was carried out by the Institute for Infectious Diseases and Zoonoses, Department of Veterinary Science, LMU Munich under the direction of Prof. Dr. med. vet. Reinhard K. Straubinger, Ph.D and were approved by the government of Upper Bavaria (approving authority for animal research). According to the approval no. 5.2-1-54-2532.3-26-13 there is no conflict with animal protection law.

### Bayesian latent class models

#### Animal samples

Out of 5736 animals tested between 2012 and 2014 in the districts Ober- and Ostallgäu (Bavaria), two data subsets with test results from multiple tests run in parallel were chosen. The first dataset comprised test results from 175 animals which had been tested from December 2012 to March 2013 by the SICT test, the Bovigam® assay, culture and pathological examination. The second dataset comprised test results from 389 animals which had been tested from April 2013 to February 2014 by the SICCT test, the PCR and which have been examined pathologically. The data was collected as binary data and to some extent, for the SICT test, the SICCT test and the Bovigam® assay, also as metric data.

#### Diagnostic tests

The SICT test and the SICCT test were performed by field or official veterinarians. Examination of the test results were carried out in accordance to Commission Regulation (EC) No 1226/2002 of 8 July 2002 amending Annex B to Council Directive 64/432/EEC [15]. For the skin tests 0.1 ml of bovine respectively bovine and avian Purified Protein Derivate (PPD) (Wirtschaftsgenossenschaft Deutscher Tierärzte eG) was injected intradermally in the neck or shoulder of the cattle. For the latent class analysis the inconclusive reactors were assigned twice, once as negative reactors (standard interpretation) and once as positive reactors (severe interpretation). An inconclusive reactor means an increase of skin thickness between 2 and 4 mm (SICT test) respectively 1 and 4 mm (SICCT test) and no occurrence of clinical signs.

For the Bovigam® assay heparinized blood was taken two to 28 days (mean of 8.45 days) after the SICT test by field practitioners. The blood was sent within 6 to 7 hours at room temperature to the laboratory of the Bavarian Health and Food Safety Authority. The Bovigam® assay was carried out according to the manufacturer’s instructions. In brief, the blood samples were stimulated overnight with avian and bovine PPD. IFN-ɣ production of the lymphocytes was then determined by using a sandwich ELISA. Identification of infected animals based on the prescription in the manufacturer’s user manual for Germany. This means that the mean optical density (OD) of a sample being stimulated with bovine PPD minus the OD of the same sample stimulated with avian PPD, was greater or equal 0.1.

The pathological examinations were performed at different places (pathology of the Bavarian Health and Food Safety Authority, carcass disposal plants) by veterinarians. Attention was given to the retropharyngeal lymph nodes, lung, gut, spleen, kidneys, liver and the associated lymph nodes as well as organs or lymph nodes with pathological-anatomical changes.

For polymerase chain reaction (PCR) samples were collected during necropsy from the retropharyngeal lymph nodes, lung, gut, spleen, kidneys, liver and the associated lymph nodes. Furthermore, pieces of organs or lymph nodes with pathological-anatomical changes were taken [23]. All samples were investigated in the laboratory of the Bavarian Health and Food Safety Authority. To increase the detection of mycobacteria samples with pathological findings were homogenized. From inconspicuous samples approximately 25 mg were used for DNA extraction. PCR aiming at detecting *Mycobacterium tuberculosis* complex-pathogens was performed for each sample separately according to the *Official Collection of Methods* [1]. The targeted sequences for PCR amplification are a hypothetical helicase and the insertion element (IS) *1081* [40, 41]. According to the official guidelines, results were interpreted as positive if both target sequences were found, as inconclusive if only one target sequence or only weak PCR signals were detected and as negative if no signals were observed. In agreement with the Friedrich-Loeffler-Institute (FLI) single runs were performed for each organ / lymph node.

Bacteriological culture was performed according to the *Official Collection of Methods* [1]. Organs were cultured as aggregate samples, except for organs with macroscopic lesions, which were cultured separately. As liquid media BD BACTEC™ MGIT™ was used. Löwenstein-Jensen and Stonebrink agar slants were used as solid culture media.

#### Statistical analysis

A Bayesian latent class approach assuming no gold standard, i.e. a perfect diagnostic test without any misclassification, was performed for the three-test dataset (SICCT test, PCR, necropsy) and the four-test dataset (SICT test, Bovigam® assay, necropsy, culture). The skin tests were considered with both their standard and severe interpretation, separately. In total, for the four-test dataset, there were four sensitivities, four specificities, one prevalence and twelve two-way covariances to be estimated, leading in total to 21 unknown parameters [42]. For the three-test dataset there were three sensitivities, three specificities, one prevalence and six two-way covariances to be estimated, leading in total to 13 unknown parameters. Due to the principle of parsimony, higher order terms of covariances were not considered. The specificity of culture was fixed to “1”, assuming that no false positive test result exists. This reduces the number of parameters to be estimated for the four-test dataset. For all other estimable parameters, first uninformative beta priors (1,1) were utilized. Second, informative priors basing on expert opinion and published test accuracies [9, 10] were utilized for the sensitivities and specificities of the SICT and the SICCT test. This was done for sensitivity analysis, respectively, for setting constraints to have still an identifiable model for the three-test dataset with taking the covariances into account [42]. To incorporate the prior information beta distributions (a,b), modeled by beta buster (http://252s-weblive.vet.unimelb.edu.au:3838/users/epi/beta.buster/), were used. For the SICT test we assumed—being 95% sure—that the sensitivity is greater than 50% with a mode at 70% (a = 13,3221; b = 6,2809) and that the specificity is greater than 70% with a mode at 85% (a = 23.903, b = 5.042). Similarly, for the SICCT test we assumed that the sensitivity is greater than 45% with a mode at 65% (a = 12.1979, b = 7.0296) and the specificity is greater than 80% with a mode at 90% (a = 42.5732, b = 5.6192). The presence of conditional dependencies between tests was checked by assessing separately the impact of each covariance term compared to a covariance term set to 0 on the other estimates. Presence of conditional dependencies was assessed graphically (histograms). Additionally, to assess if higher-level conditional dependencies potentially affect the results, random effect models based on the model from Qu et al. 1996 were also explored using the R package randomLCA [43, 44].

Model selection was based on DIC (Deviance Information Criterion) with lower values indicating a better model fit. For a sensitivity analysis of the three-test dataset considering the covariances uninformative priors were used. The best fitting model of the four-test dataset was additionally run with a higher cut off of the Bovigam® assay (OD difference 0.2 instead of 0.1). Due to missing values for continuous Bovigam® assay results, only 171 animals could be included. The models were implemented in JAGS (Just Another Gibbs Sampler) version 3.4.0 for Markov Chain Monte Carlo Simulation (http://mcmc-jags.sourceforge.net/), the software R version 3.0.3 (https://www.r-project.org/) and the package coda [45]. The model code is given in the supplementary online material (S1 Text). For all models the first 20,000 iterations were discarded as burn-in and based on the next 200,000 iterations the posterior distributions of the unknown parameters were derived. Three chains were run from different starting points. Convergence was checked visually by inspecting the density plots of the three chains.

The positive and negative predictive values of the skin tests (standard and severe interpretation), Bovigam® assay (OD difference 0.1 and 0.2), PCR, necropsy and culture were derived based on the estimated prevalence and posteriors obtained from the different models.

### Kappa test of agreement

#### Animal samples and testing

Blood was taken from 21 cows (Braunvieh breed) at six different time points from the *V*. *jugularis*. All animals belonged to one farm and were tested previously as positive or inconclusive with the SICT test. On two time points the blood was additionally taken from the *V*. *caudalis mediana* resp. *V*. *subcutanea abdominis*. Immediately after collection, the blood was sent to five laboratories, all over Germany. After arriving at the laboratories the blood was directly examined with the Bovigam® assay. Due to the fact that the laboratories were distributed all over Germany the time between blood collection and further examination was between 4 to 29 h with a median of 8.0 h. The samples were not blinded.

#### Statistical analysis

To determine if the laboratories were classifying approximately the same proportion of individuals as positive, first McNemar's test was applied for each given time point between all possible pairwise laboratory comparisons [46]. McNemar's test was performed with the software R version 3.0.3 (https://www.r-project.org/) with the package exact2x2 [47]. For the inter-laboratory agreement the time point with the best accordance of the proportion of positive test results was chosen to determine Cohen's kappa. Also the agreement between the test results of the Bovigam® assay from the differing localizations was estimated using McNemar's test and Cohen's kappa. Cohen's kappa was calculated online with Graphpad software (http://graphpad.com/quickcalcs/kappa2/).

## Results

### Bayesian latent class models

The raw data, comprising the dichotomized test results of the four-test and the three-test dataset, are presented in S1 and S2 Tables.

#### Four-test dataset

Posterior means and corresponding 95% credibility intervals resulting from Bayesian latent class models are shown in Table 1.

**Table 1:** Prevalence and diagnostic test accuracies of different models considered from the dataset with 175 animals tested with SICT test, Bovigam® assay, culture [sp = 100%] and necropsy.

Model	Prevalence (95% CI)	SICT test (95% CI)		Bovigam® assay (95% CI)		Culture (95% CI)		Necropsy (95% CI)	
		se	sp	se	sp	se	sp	Se	sp
**1**	**7.7 (4.2–12.3)**	**70.3 (44.9–90.5)**	**75.8 (68.8–82.2)**	**95.7 (91.3–99.2)**	**6.9 (3.6–11.1)**	**88.9 (65.5–99.7)**	**fixed at 100**	**76.8 (51.6–94.4)**	**99.0 (96.8–100)**
2	7.7 (4.2–12.3)	70.1 (53.5–84.7)	77.1 (70.9–82.8)	95.8 (91.3–99.2)	6.9 (3.6–11.1)	88.9 (65.0–99.7)	fixed at 100	76.9 (51.7–94.4)	98.9 (96.8–99.9)
3	7.9 (4.3–12.6)	70.2 (45.1–90.4)	76.4 (69.7–82.6)	83.3 (74.2–93.5)	23.5 (17.4–30.3)	88.9 (64.8–99.7)	fixed at 100	77.0 (51.2–94.6)	98.9 (96.7–99.9)
4	7.9 (4.3–12.5)	98.2 (94.9–99.9)	4.1 (1.7–7.5)	95.7 (91.2–99.2)	6.9 (3.7–11.2)	87.5(62.8–99.5)	fixed at 100	77.1 (51.4–94.6)	99.1 (97.0–100)

Model 1: SICT test [standard interpretation, uninformative priors], Bovigam® assay [cut-off = 0.1], culture [sp = 100%], no covariances

Model 2: SICT test [standard interpretation, informative priors], Bovigam® assay [cut-off = 0.1], culture [sp = 100%], no covariances

Model 3: SICT test [standard interpretation, uninformative priors], Bovigam® assay [cut-off = 0.2], culture [sp = 100%], no covariances

Model 4: SICT test [severe interpretation, uninformative priors], Bovigam® assay [cut-off = 0.1], culture [sp = 100%] no covariances

se, sensitivity

sp, specificity

There was no evidence, based on DIC and visual inspection of covariance histograms that including any covariance term led to a better model fit (S3 Table and S1 Fig). Including covariance terms did also not alter the posterior means. Adding random effects to model higher level conditional dependencies did not improve model fit.

If informative instead of flat priors were used for SICT test the DIC decreased slightly (392.6 instead of 393.7) and the posterior means were only marginally affected. If a higher cut-off of 0.2 instead of 0.1 for the Bovigam® assay was applied, then the sensitivity of the Bovigam® assay decreased from 95.7% (91.3‒99.2%) to 83.3% (74.2‒93.5%) and the specificity increased from 6.9% (3.6‒11.1%) to 23.5% (17.4‒30.3%). The estimated posteriors of the other tests and the prevalence differed maximally around 0.4%. The dichotomized test results are presented in S4 Table. When interpreting the inconclusive test results of the SICT test as positive the specificity of the SICT test was extremely low with 4.1% (1.7‒7.5%).

The positive and negative predictive values for the SICT test (standard and severe interpretation), Bovigam® assay (cut-off 0.1 and 0.2), necropsy and culture are presented in S5 Table.

#### Three-test dataset

The posterior sensitivities and specificities for the three-test dataset resulting from the Bayesian latent class models are presented in Table 2. With the incorporated informative priors the sensitivity of the SICCT test increased by 2%. The estimated test characteristics of the other tests were only marginally affected. With regard to the histograms (S2 Fig), the posteriors and the DIC (S6 Table) dependence between the sensitivity of the PCR and necropsy seemed to be the most likely. Within this model the sensitivities of the PCR and the necropsy decreased and the prevalence increased. The remaining posteriors range around the same values.

**Table 2:** Prevalence and diagnostic test accuracies of different models considered from the dataset with 389 animals tested with SICCT test [standard interpretation], PCR and necropsy.

Model	Prevalence (95% CI)	SICCT test (95% CI)		PCR (95% CI)		Necropsy (95% CI)	
		se	sp	se	sp	se	sp
1	17.3 (13.5–21.5)	55.5 (43.3–67.7)	91.7 (88.3–94.6)	80.6 (69.1–90.6)	99.2 (97.6–100)	90.6 (80.6–98.0)	99.1 (97.2–100)
2	17.2 (13.4–21.4)	57.5 (46.5–68.1)	91.5 (88.4–94.2)	80.6 (69.1–90.6)	99.1 (97.5–100)	90.7 (80.7–98.0)	99.1 (97.2–100)
**3**	**19.8 (14.6–26.5)**	**57.8 (48.0–67.6)**	**92.8 (89.2–96.3)**	**70.6 (52.0–86.0)**	**99.0 (97.4–99.9)**	**78.4 (58.6–93.7)**	**98.9 (96.8–100)**
4	21.8 (15.1–30.9)	56.1 (46.3–66.2)	94.5 (89.7–99.5)	65.2 (44.6–84.5)	99.0 (97.4–99.9)	72.4 (49.8–92.5)	99.0 (96.9–100)
5	15.6 (11.4–20.2)	94.9 (89.5–98.8)	12.0 (8.7–15.8)	87.9 (73.0–99.4)	98.8 (96.9–100.0)	92.2 (81.3–99.6)	97.6 (94.6–99.9)

Model 1: SICCT test [standard interpretation, uninformative priors], no covariances

Model 2: SICCT test [standard interpretation, prior information of se and sp], no covariances

Model 3: SICCT test [standard interpretation, prior information of se and sp], covariance between sensitivity PCR and necropsy

Model 4: SICCT test [standard interpretation, uninformative priors], covariance between sensitivity PCR and necropsy

Model 5: SICCT test [severe interpretation, uninformative priors], no covariances

se, sensitivity

sp, specificity

By running this model with flat priors the DIC increased to 754.8 instead of 750.7 and the sensitivities of PCR and necropsy decreased around 5.4% and 6.0%. The other estimated parameters differed maximal around 2%. The density plots of the estimated probability distributions showed better convergence for the model with the SICCT test as standard interpretation. The specificity for the SICCT test was extremely low with 12.0% (8.7‒15.8%) for the severe interpretation.

The positive and negative predictive values for the SICCT test (standard and severe interpretation), PCR and necropsy based on the estimated posteriors and prevalence of the three test data set can be found in S7 Table.

### Inter- and intra-laboratory agreement

The raw data utilized for assessing agreement between the different laboratories and the different localizations are presented in S8 and S9 Tables. Based on McNemar's test to assess if the proportions of samples classified as positive differed significantly between the laboratories or the anatomical location, the time point with most non-significant tests was chosen (Table 3) [46]. Estimated *p-*values for McNemar's test ranged from 0.03 to 1.00. The inter-laboratory agreement between Laboratory 2 and 3 reached a Cohen's kappa value of 0.62 (95% confidence interval from 0.15 to 1.00). The other agreements constituted between -0.16 (95% confidence interval from -0.32 to -0.01) and 0.38 (95% confidence interval from 0.01 to 0.76).

**Table 3:** Comparison of the Bovigam® assay test results from five different laboratories by the calculated *p*-value based on McNemar's test, Cohen's kappa values with the 95% confidence interval and the proportions of the test results of one given time point.

Comparison between:	*p*-value	Kappa	CI	Proportions of test results^a^		
				pos/pos	neg/neg	disconcordant
Lab 1 / Lab 2	**0.69**	**0.31**	-0.12 to 0.74	57%	14%	29%
Lab 1 / Lab 3	**0.38**	**0.30**	-0.15 to 0.74	63%	11%	26%
Lab 1 / Lab 4	**1.00**	**0.26**	-0.23 to 0.75	61%	11%	28%
Lab 1 / Lab 5	**0.75**	**0.00**	-0.42 to 0.42	38%	14%	48%
Lab 2 / Lab 3	**1.00**	**0.83**	0.50 to 1.00	79%	16%	5%
Lab 2 / Lab 4	**1.00**	**0.26**	-0.23 to 0.75	61%	11%	28%
Lab 2 / Lab 5	**0.22**	**0.38**	0.01 to 0.76	52%	19%	29%
Lab 3 / Lab 4	**1.00**	**0.09**	-0.42 to 0.61	63%	6%	31%
Lab 3 / Lab 5	**0.06**	**0.41**	0.05 to 0.77	58%	16%	26%
Lab 4 / Lab 5	**0.45**	**0.11**	-0.33 to 0.55	50%	11%	39%

Lab, Laboratory; neg, negative; pos, positive; CI, 95% confidence interval

^a^The number of analyzable test results ranged from 16–21 animals

For the agreement between the varying localizations nearly all McNemar's tests are non-significant (Table 4), thus indicating the proportion of positive test results did not significantly differ between the laboratories. One estimated *p*-value based on McNemar's test was 0.03, giving evidence that there is a disagreement between the two proportions of Bovigam® test results [46]. The best agreement was seen for Laboratory 3 by comparing the Bovigam® assay results between the blood of the *V*. *jugularis* and *V*. *subcutanea abdominis* (1.00). Also the agreement between the results of the *V*. *jugularis* and the *V*. *caudalis mediana* of this laboratory reached at least a Cohen's kappa value of 0.62 (95% confidence interval from 0.00 to 1.00). Laboratory 5 had a substantial agreement by the comparison between *V*. *jugularis* and *V*. *caudalis mediana*. All other agreements were below 0.54 indicating a poor to moderate agreement.

**Table 4:** Comparison of the Bovigam® assay test results between differing localizations by calculating *p*-values based on McNemar's test and Cohen's kappa values.

		Lab 1	Lab 2	Lab 3	Lab 4	Lab 5
*V*.*j*. */ V*.*s*.*a*.	***p*-value**	**1.00**	**0.13**	**1.00**	**0.38**	**0.03**
	**kappa**	**0.29**	**0.41**	**1.00**	**0.46**	**0.37**
	CI	-0.12‒0.72	0.00‒0.86	1.00‒1.00	0.03‒0.83	0.08‒0.74
*V*.*j*. */ V*.*c*.*m*.	***p*-value**	**0.45**	**0.63**	**0.50**	**1.00**	**0.25**
	**kappa**	**0.16**	**0.54**	**0.62**	**-0.03**	**0.70**
	CI	-0.22‒0.59	0.03‒0.90	0.00‒1.00	-0.37‒0.43	0.36‒1.00

*V*.*j*.: *V*. *jugularis; V*.*s*.*a*.: *V*. *subcutanea abdominis; V*.*c*.*m*.: *V*. *caudalis mediana*; CI: 95% confidence interval

## Discussion

Due to the detection of bovine tuberculosis at several occasions during regular abattoir meat inspections in the Allgäu region, a new tuberculosis control program was implemented in November 2012 in Germany. Within this testing regime the Bovigam® assay was performed for the first time as a field test in Germany. The test results gained from this control program were utilized to estimate the sensitivities and specificities of the different tests with a latent class analysis. This was especially of interest as the persons involved in this testing program recognized contradictory test outcomes for the Bovigam® assay which led to distrust and termination of the testing with the Bovigam® assay. These contradictory test outcomes seem to be corroborated by the raw data presented in S1 Table, where out of 175 tested animals 115 were diagnosed positive only with the Bovigam® assay.

The estimated test characteristics of the SICT and the SICCT test, the PCR, necropsy and culture are in line with already published data [9, 10, 13, 28, 31, 48, 49]. For the Bovigam® assay an extremely low specificity was estimated. In this population with an estimated true prevalence of 7.7 the positive predictive values of the Bovigam® assay would be 7.9% (OD difference of 0.1) respectively 8.54% (OD difference of 0.2).

This finding could be corroborated with additional intra- and inter-laboratory testing of agreement.

In this study no-gold-standard-models, relying on Bayesian latent class approaches, which are increasingly used in medical and veterinary sciences, were used [50, 51]. The specificity of the culture was set at 100%, as a positive result is assumed to be truly positive [7]. The best fitting model was chosen by DIC. Additionally, as the DIC has its limitations, histograms and posteriors were evaluated [52–54]. In order to comply with good statistical practice all possible two way covariances were taken into account [55]. A conditional dependency could only be seen between the sensitivity of PCR and necropsy. These two examination methods do not rely on similar biological basics, but were related as sample selection and sample size for the PCR were associated with pathological examination. Due to the fact that both datasets were quite small, conditional dependence between other tests could not be excluded. Inclusion of prior information of the SICT test, for a sensitivity analysis of the four-test dataset, did not influence the posteriors. For the three-test dataset the DIC increases by running a sensitivity analysis with flat priors, indicating a worse model fit. As already shown by Álvarez et al. [10] the test characteristics of the skin tests alter with a severe interpretation insofar that the sensitivity increases and the specificity decreases. Within our data a strong shift to lower specificities could be seen for the severe interpretation of the skin tests. This outcome seems to be data driven, as in both datasets most of the animals had an inconclusive skin test result. Therefore and because of the poorer convergence for the models with severe interpretation, which could be due to the small amount of true positive test results, the focus was set on the skin tests standard interpretation.

For the skin tests, PCR, necropsy and culture, the estimated sensitivities and specificities are in accordance of test characteristics from other publications [9, 10, 13, 28, 31, 48, 49]. The wide credibility intervals for the sensitivities (19.6 to 45.6) could be explained by the small data pool of true positive animals. With regard to the estimated test characteristics of the skin tests it has to be considered that these could have been affected by the performance of the skin tests [56]. The test characteristics of the pathological examination were within both datasets 76.8% (51.6–94.4%) and 78.4% (58.6–93.7%), respectively, for the sensitivity and around 99.0% (96.8–100%) for the specificity. The fact that the pathological examination was done in different localizations from different persons as well as the small data pool of true positive animals explains the wide credibility interval for the sensitivity. This spectrum bias appears to be present in both subpopulations. Within our estimated test characteristics the SICCT test is less sensitive although more specific than the SICT test. PCR and necropsy are less sensitive than culture. Therefore, culture is still an essential diagnostic tool.

In the literature the test characteristics of the Bovigam® assay are stated as between 66.9–100% for sensitivity and 70–99.6% for specificity [9, 12, 49]. We estimated a quite high sensitivity of 95.7% (91.3–99.2%), but an extremely low specificity of 6.9% (3.6–11.1%). This stands in line with the experience of the persons involved in the bovine TB testing. With setting the cut-off higher an increase in the specificity was expected, as already reported by others [57, 58]. Indeed, the specificity raised to 23.5% (17.4–30.3%) thereby the sensitivity decreased to 83.3% (74.2–93.5%), This shows again that the model itself is robust. To our knowledge such low specificities were never recognized before for the Bovigam® assay. Although it was already stated that the Bovigam® assay is more sensitive, but less specific than the SICCT test [59]. And it was shown that fewer than 20% of the animals tested positive in the Bovigam® assay were also positive in culture or pathology [60]. Van Dijk [61] showed that the Bovigam® assay is likely to have false positive results and this in a higher amount than the SICCT test. With a decrease of the prevalence the amount of false positive test results even increases [61].

Among the influential factors discussed in literature a previously performed skin test is discussed to have an effect on the specificity of the Bovigam® assay. Within our study the SICT test was performed two to 28 (mean of 8.45 days) days prior to the Bovigam® assay. Several studies discuss the effect of a previous skin test (either CFT test or SICCT test) towards the IFN-ɣ production in natural or experimental infected cattle. Whereas the CFT test leads to a clear increase of IFN-ɣ production, this influence is not obvious after the SICCT test [62]. The previously performed skin test in this study may have had an impact on the estimated specificity within the examined subpopulation. A genetic influence and an association between the breed and the outcome of the SICCT test were reported by Amos et al. [63]. This was not seen for the Bovigam® assay [64]. An influence of breed and genetics might be present in the Allgäu region, but further investigations have to be made to confirm this. The Bovigam® assay was only carried out within the regions Ober- and Ostallgäu during November 2012 until March 2013. This regional and seasonal limitation could have had an impact on the high amount of false positive test results [64, 65]. The correlation between season and occurrence of saprophytic mycobacteria might be associated with this [66, 67]. Moreover, infections with *Mycobacterium avium* subspecies *paratuberculosis* (MAP) may lead to false positive results for the Bovigam® assay [68]. Since the tested animals have not been examined for a concurrent MAP infection this impact could not be excluded. Furthermore, an infection with *Fasciola hepatica* is also reported to influence the IFN-ɣ response. Although this is until now only stated for the skin test and in context of false negative test results [69]. The specificity of the Bovigam® assay tests varies also with the concentration and potency of PPDs [70], which can differ remarkably [71, 72]. These influences might explain to some extend the estimated low specificities of the Bovigam® assay. Besides, the fact that bovine tuberculosis, in the regions Ober- and Ostallgäu was caused by *Mycobacterium caprae* may have influenced the Bovigam® assay results, too, as bovine tuberculosis in other regions worldwide is predominantly caused by *Mycobacterium bovis*. However, the low inter- and intralaboratory agreements between the Bovigam® assay outcomes could not be fully explained by this. The transportation time as well as the experience seems to influence the Bovigam® assay test outcomes, as between the laboratories with the shortest transportation time (Laboratory 3, data not shown) and the most experience (Laboratory 2 and 3, data not shown) the best, but still only substantial agreement was estimated. By comparing the intralaboratory agreement between the different laboratories again the laboratory with the most experience and the shortest transportation time (Laboratory 3) had the best agreement between the results of the blood taken from the *V*. *jugularis* and the *V*. *subcutanea abdominis*. A longer storage or transportation of the blood samples might lead to a decrease in the mean OD or the IFN-ɣ production [73–75]. With regard to the sensitivity and specificity of the Bovigam® assay several studies state that blood could also be processed 24 h later without statistical significant changes [76, 77]. However, Laboratory 3 reached also only a substantial agreement of 0.62 between the Bovigam® assay test results of the *V*. *jugularis* and the *V*. *caudalis mediana*. As for the intralaboratory agreement only the localization of the blood collection altered, much better accordance between the Bovigam® assay test results were expected, as blood taken from differing localizations should not differ [78, 79]. But the smaller diameter of the tail vain could lead to more damage and therefore micro-clotting, resulting in captured lymphocytes and therefore lower IFN-ɣ release [80]. Despite, there are conflicting views if an equal distribution of all lymphocyte subpopulations all over the body can be assumed in general. Regarding this, it must be taken into account that a blood sample can only give a snapshot. Although all five laboratories were officially approved none of them reported good concordance for the Bovigam® assay test results. It seems that the Bovigam® assay is a diagnostic tool with some disadvantages. Many influences including external factors (MAP, saprophytic mycobacteria, previous skin test and genetic components) and factors directly connected with the test performance, as the concentration of the PPDs, transportation time of the blood, localization of blood collection and also the experience of the laboratories might lead to differing test results. A higher specificity of the Bovigam® assay, especially in low prevalence herds and animals having a co-infection with MAP, can be achieved by using the proteins ESAT6 and CFP10 instead of PPDa and PPDb [68, 81]. Also working out and evaluating an individual test performance (proteins, protein concentration, cut-off etc.) for each Bovigam® assay application as a field test might lead to better test characteristics [64, 70]. This could be demonstrated in this study in so far as the specificity increased by setting the cut-off to 0.2.

To our knowledge the use of a latent class analysis for the estimation of test characteristics for bTB diagnostic tests was never done before in Germany. An important strength of this study is that the data were gained from surveillance and therefore originates from a special epidemiologic situation. But this means also that only a subpopulation was tested and the animals were not chosen randomly. According to this background information our findings cannot be generalized. Additionally a new version of the Bovigam® assay has been developed since 2013 to which our findings cannot be transferred [82].

## Conclusion

With this latent class analysis the test characteristics of different diagnostic tests used in the current bovine TB outbreak in Southern Germany could be estimated. Within this study an extremely low specificity and a low inter- and intralaboratory agreement were estimated for the Bovigam® assay. These findings might be due to influences affecting the environment or the immune system of the cow. Also factors that are associated with the testing procedure and the laboratories chosen might have had an effect. Therefore, the change during the testing regime towards SICCT test as only ante-mortem test was correct and founded. Despite the fact that the Bovigam® assay has been further advanced, a previous test evaluation prior to future surveillance programs is highly recommended. The estimated test characteristics for the other tests were in an acceptable range.

## Supporting information

S1 Text Bayesian latent-class model code for four diagnostic tests. (DOCX)

S1 Table Number of test result combinations in the four-test dataset (n = 175), the inconclusive test results of the SICT test once considered as negative (standard interpretation) and once as positive (severe interpretation). ^b^ cut-off: 0.1.
(DOCX)

S2 Table Number of test result combinations in the three-test dataset (n = 389), the inconclusive test results of the SICCT test once considered as negative (standard interpretation) and once as positive (severe interpretation). (DOCX)

S3 Table DIC, prevalence and diagnostic test accuracies of different models, without and with covariances of the sensitivities between the different tests, considered from the dataset (n = 175) tested with SICT test [standard interpretation, uninformative priors], Bovigam® assay [cut-off = 0.1], culture [sp = 100%] and necropsy. Model 1: no covariances; Model 2: covariance sensitivity SICT test, Bovigam® assay; Model 3: covariance sensitivity SICT test, culture; Model 4: covariance sensitivity SICT test, pathology; Model 5: covariance sensitivity Bovigam® assay, culture; Model 6: covariance sensitivity Bovigam® assay, pathology; Model 7: covariance sensitivity culture, pathology; CI, credibility interval; se, sensitivity; sp, specificity.
(DOCX)

S4 Table Dichotomized test results of the Bovigam® assay for two different cut-offs. (DOCX)

S5 Table Positive and negative predictive values of the SICT test, Bovigam® assay, culture [sp = 100%] and necropsy calculated from the prevalence and diagnostic test accuracies obtained from the models of Table 1. PPV, positive predictive value; NPV, negative predictive value.
(DOCX)

S6 Table DIC, prevalence and diagnostic test accuracies of different models, without and with covariances of the sensitivities and specificities between the different tests, considered from the dataset (n = 389) tested with SICCT test [standard interpretation; prior information], PCR and necropsy. Model 1: no covariances; Model 2: covariance sensitivity SICCT test, PCR; Model 3: covariance sensitivity SICCT test, pathology; Model 4: covariance sensitivity PCR, pathology; Model 5: covariance specificity SICCT test, PCR; Model 6: covariance specificity SICCT test, pathology; Model 7: covariance specificity PCR, pathology; CI, credibility interval; se, sensitivity; sp, specificity.
(DOCX)

S7 Table Positive and negative predictive values of the SICCT test, PCR and necropsy calculated from the prevalence and diagnostic test accuracies obtained from the models of Table 2. PPV, positive predictive value; NPV, negative predictive value.
(DOCX)

S8 Table Test results of the Bovigam® assay from five officially approved laboratories; the results of the time point with the best accordance of the proportion of positive test results is shown. Pos, positive; neg, negative; n.a., not analyzable.
(DOCX)

S9 Table Test results of the Bovigam® assay from five officially approved laboratories. **The assayed blood was taken at two time points respectively from two differing localizations.** *V*.*j*., *V*. *jugularis; V*.*s*.*a*., *V*. *subcutanea abdominis; V*.*c*.*m*., *V*. *caudalis mediana*; pos, positive; neg, negative;?, inconclusive; n.a., not analyzable.
(DOCX)

S1 Fig Histograms from the covariances of the sensitivities between the different tests, considered from the four-test dataset (n = 175) tested with SICT test [standard interpretation; no prior information], Bovigam® assay [cut−off = 0.1], culture [sp = 100%] and necropsy. (PDF)

S2 Fig Histograms from the covariances of the sensitivities and specificities between the different tests, considered from the three-test dataset (n = 389) tested with SICCT test [standard interpretation; prior information], PCR and necropsy. (PDF)
