# Supplementary material for: Evaluating diagnostic tests for bovine tuberculosis in the southern part of Germany: A latent class analysis
Source: PLoS One. 2017 Jun 22;12(6):e0179847. doi: 10.1371/journal.pone.0179847 (PMC5481003; doi:10.1371/journal.pone.0179847)
Supplement: S1 Text — (DOCX) [file pone.0179847.s001.docx]

**S1 Text: Bayesian latent-class model code for four diagnostic tests**

#######################################################

##Definition of the variables in the model

#######################################################

var p[N], q[N,8], pr[N], L[N],checks[N,16];

#N <- observations

# p <- individual samples

# q <- different combinations of test results

# pr <- prevalence

# s <- test sensitivities

# c <- test specificities

# cs <- conditional dependency between tests sensitivities

# cc <- conditional dependency between tests specificities

# mobokupa <- data set name

#######################################################

## Modelling the different probabilities of combinations of tests results

#######################################################

model {

for(i in 1:N){

q[i,1]<-pr[i]*(s1*s2*s3*s4+cs12+cs13+cs23+cs14+cs24+cs34)+(1-pr[i])*((1-c1)*(1-c2)*(1-c3)*(1-c4)+cc12+cc13+cc23+cc14+cc24+cc34);

q[i,2]<-pr[i]*(s1*s2*s3*(1-s4)+cs12+cs13+cs23-cs14-cs24-cs34)+(1-pr[i])*((1-c1)*(1-c2)*(1-c3)*c4+cc12+cc13+cc23-cc14-cc24-cc34);

q[i,3]<-pr[i]*(s1*s2*(1-s3)*s4+cs12-cs13-cs23+cs14+cs24-cs34)+(1-pr[i])*((1-c1)*(1-c2)*c3*(1-c4)+cc12-cc13-cc23+cc14+cc24-cc34);

q[i,4]<-pr[i]*(s1*s2*(1-s3)*(1-s4)+cs12+cs13-cs23-cs14-cs24+cs34)+(1-pr[i])*((1-c1)*(1-c2)*c3*c4-cc12+cc13-cc23-cc14-cc24+cc34);

q[i,5]<-pr[i]*(s1*(1-s2)*s3*s4-cs12+cs13-cs23+cs14-cs24+cs34)+(1-pr[i])*((1-c1)*c2*(1-c3)*(1-c4)-cc12+cc13-cc23+cc14-cc24+cc34);

q[i,6]<-pr[i]*(s1*(1-s2)*s3*(1-s4)-cs12+cs13-cs23-cs14+cs24-cs34)+(1-pr[i])*((1-c1)*c2*(1-c3)*c4-cc12+cc13-cc23-cc14+cc24-cc34);

q[i,7]<-pr[i]*(s1*(1-s2)*(1-s3)*s4-cs12-cs13+cs23+cs14-cs24-cs34)+(1-pr[i])*((1-c1)*c2*c3*(1-c4)-cc12-cc13+cc23+cc14-cc24-cc34);

q[i,8]<-pr[i]*(s1*(1-s2)*(1-s3)*(1-s4)-cs12-cs13+cs23-cs14+cs24+cs34)+(1-pr[i])*((1-c1)*c2*c3*c4-cc12-cc13+cc23-cc14+cc24+cc34);

q[i,9]<-pr[i]*((1-s1)*s2*s3*s4-cs12-cs13+cs23-cs14+cs24+cs34)+(1-pr[i])*(c1*(1-c2)*(1-c3)*(1-c4)-cc12-cc13+cc23-cc14+cc24+cc34);

q[i,10]<-pr[i]*((1-s1)*s2*s3*(1-s4)-cs12-cs13+cs23+cs14-cs24-cs34)+(1-pr[i])*(c1*(1-c2)*(1-c3)*c4-cc12-cc13+cc23+cc14-cc24-cc34);

q[i,11]<-pr[i]*((1-s1)*s2*(1-s3)*s4-cs12+cs13-cs23-cs14+cs24-cs34)+(1-pr[i])*(c1*(1-c2)*c3*(1-c4)-cc12+cc13-cc23-cc14+cc24-cc34);

q[i,12]<-pr[i]*((1-s1)*s2*(1-s3)*(1-s4)-cs12+cs13-cs23+cs14-cs24+cs34)+(1-pr[i])*(c1*(1-c2)*c3*c4-cc12+cc13-cc23+cc14-cc24+cc34);

q[i,13]<-pr[i]*((1-s1)*(1-s2)*s3*s4+cs12-cs13-cs23-cs14-cs24+cs34)+(1-pr[i])*(c1*c2*(1-c3)*(1-c4)+cc12-cc13-cc23-cc14-cc24+cc34);

q[i,14]<-pr[i]*((1-s1)*(1-s2)*s3*(1-s4)+cs12-cs13-cs23+cs14+cs24-cs34)+(1-pr[i])*(c1*c2*(1-c3)*c4+cc12-cc13-cc23+cc14+cc24-cc34);

q[i,15]<-pr[i]*((1-s1)*(1-s2)*(1-s3)*s4+cs12+cs13+cs23-cs14-cs24-cs34)+(1-pr[i])*(c1*c2*c3*(1-c4)+cc12+cc13+cc23-cc14-cc24-cc34);

q[i,16]<-pr[i]*((1-s1)*(1-s2)*(1-s3)*(1-s4)+cs12+cs13+cs23+cs14+cs24+cs34)+(1-pr[i])*(c1*c2*c3*c4+cc12+cc13+cc23+cc14+cc24+cc34);

#######################################################

## Check and correct potential errors of probabilities exceeding (0,1) bounds

#######################################################

checks[i,1]<- s1*s2*s3*s4+cs12+cs13+cs23+cs14+cs24+cs34;

checks[i,2]<- (1-c1)*(1-c2)*(1-c3)*(1-c4)+cc12+cc13+cc23+cc14+cc24+cc34;

checks[i,3]<- s1*s2*s3*(1-s4)+cs12+cs13+cs23-cs14-cs24-cs34;

checks[i,4]<- (1-c1)*(1-c2)*(1-c3)*c4+cc12+cc13+cc23-cc14-cc24-cc34;

checks[i,5]<- s1*s2*(1-s3)*s4+cs12-cs13-cs23+cs14+cs24-cs34;

checks[i,6]<- (1-c1)*(1-c2)*c3*(1-c4)+cc12-cc13-cc23+cc14+cc24-cc34;

checks[i,7]<- s1*s2*(1-s3)*(1-s4)+cs12+cs13-cs23-cs14-cs24+cs34;

checks[i,8]<- (1-c1)*(1-c2)*c3*c4-cc12+cc13-cc23-cc14-cc24+cc34;

checks[i,9]<- s1*(1-s2)*s3*s4-cs12+cs13-cs23+cs14-cs24+cs34;

checks[i,10]<- (1-c1)*c2*(1-c3)*(1-c4)-cc12+cc13-cc23+cc14-cc24+cc34;

checks[i,11]<- s1*(1-s2)*s3*(1-s4)-cs12+cs13-cs23-cs14+cs24-cs34;

checks[i,12]<- (1-c1)*c2*(1-c3)*c4-cc12+cc13-cc23-cc14+cc24-cc34;

checks[i,13]<- s1*(1-s2)*(1-s3)*s4-cs12-cs13+cs23+cs14-cs24-cs34;

checks[i,14]<- (1-c1)*c2*c3*(1-c4)-cc12-cc13+cc23+cc14-cc24-cc34;

checks[i,15]<- s1*(1-s2)*(1-s3)*(1-s4)-cs12-cs13+cs23-cs14+cs24+cs34;

checks[i,16]<- (1-c1)*c2*c3*c4-cc12-cc13+cc23-cc14+cc24+cc34;

checks[i,17]<- (1-s1)*s2*s3*s4-cs12-cs13+cs23-cs14+cs24+cs34;

checks[i,18]<- c1*(1-c2)*(1-c3)*(1-c4)-cc12-cc13+cc23-cc14+cc24+cc34;

checks[i,19]<- (1-s1)*s2*s3*(1-s4)-cs12-cs13+cs23+cs14-cs24-cs34;

checks[i,20]<- c1*(1-c2)*(1-c3)*c4-cc12-cc13+cc23+cc14-cc24-cc34;

checks[i,21]<- (1-s1)*s2*(1-s3)*s4-cs12+cs13-cs23-cs14+cs24-cs34

checks[i,22]<- c1*(1-c2)*c3*(1-c4)-cc12+cc13-cc23-cc14+cc24-cc34;

checks[i,23]<- (1-s1)*s2*(1-s3)*(1-s4)-cs12+cs13-cs23+cs14-cs24+cs34;

checks[i,24]<- c1*(1-c2)*c3*c4-cc12+cc13-cc23+cc14-cc24+cc34;

checks[i,25]<- (1-s1)*(1-s2)*s3*s4+cs12-cs13-cs23-cs14-cs24+cs34;

checks[i,26]<- c1*c2*(1-c3)*(1-c4)+cc12-cc13-cc23-cc14-cc24+cc34;

checks[i,27]<- (1-s1)*(1-s2)*s3*(1-s4)+cs12-cs13-cs23+cs14+cs24-cs34;

checks[i,28]<- c1*c2*(1-c3)*c4+cc12-cc13-cc23+cc14+cc24-cc34;

checks[i,29]<- (1-s1)*(1-s2)*(1-s3)*s4+cs12+cs13+cs23-cs14-cs24-cs34;

checks[i,30]<- c1*c2*c3*(1-c4)+cc12+cc13+cc23-cc14-cc24-cc34;

checks[i,31]<- (1-s1)*(1-s2)*(1-s3)*(1-s4)+cs12+cs13+cs23+cs14+cs24+cs34;

checks[i,32]<- c1*c2*c3*c4+cc12+cc13+cc23+cc14+cc24+cc34;

valid[i]<- step(s1+c1-1.0)*step(s2+c2-1.0)*step(s3+c3-1.0)*step(s4+c4-1.0)*

step(1-checks[i,1])*step(checks[i,1])*

step(1-checks[i,2])*step(checks[i,2])*

step(1-checks[i,3])*step(checks[i,3])*

step(1-checks[i,4])*step(checks[i,4])*

step(1-checks[i,5])*step(checks[i,5])*

step(1-checks[i,6])*step(checks[i,6])*

step(1-checks[i,7])*step(checks[i,7])*

step(1-checks[i,8])*step(checks[i,8])*

step(1-checks[i,9])*step(checks[i,9])*

step(1-checks[i,10])*step(checks[i,10])*

step(1-checks[i,11])*step(checks[i,11])*

step(1-checks[i,12])*step(checks[i,12])*

step(1-checks[i,13])*step(checks[i,13])*

step(1-checks[i,14])*step(checks[i,14])*

step(1-checks[i,15])*step(checks[i,15])*

step(1-checks[i,16])*step(checks[i,16])*

step(1-checks[i,17])*step(checks[i,17])*

step(1-checks[i,18])*step(checks[i,18])*

step(1-checks[i,19])*step(checks[i,19])*

step(1-checks[i,20])*step(checks[i,20])*

step(1-checks[i,21])*step(checks[i,21])*

step(1-checks[i,22])*step(checks[i,22])*

step(1-checks[i,23])*step(checks[i,23])*

step(1-checks[i,24])*step(checks[i,24])*

step(1-checks[i,25])*step(checks[i,25])*

step(1-checks[i,26])*step(checks[i,26])*

step(1-checks[i,27])*step(checks[i,27])*

step(1-checks[i,28])*step(checks[i,28])*

step(1-checks[i,29])*step(checks[i,29])*

step(1-checks[i,30])*step(checks[i,30])*

step(1-checks[i,31])*step(checks[i,31])*

step(1-checks[i,32])*step(checks[i,32]);

#######################################################

## Contribution to the likelihood for each observation

#######################################################

L[i]<- equals(valid[i],1)*(

equals(mobokupa [i,2],1)*equals(mobokupa[i,3],1)*equals(mobokupa [i,4],1)*equals (mobokupa [i,6],1)*q[i,1]

+ equals(mobokupa [i,2],1)*equals(mobokupa[i,3],1)*equals(mobokupa [i,4],1)*equals (mobokupa [i,6],0)*q[i,2]

+ equals(mobokupa [i,2],1)*equals(mobokupa[i,3],1)*equals(mobokupa [i,4],0)*equals (mobokupa [i,6],1)*q[i,3]

+ equals(mobokupa [i,2],1)*equals(mobokupa[i,3],1)*equals(mobokupa [i,4],0)*equals (mobokupa [i,6],0)*q[i,4]

+ equals(mobokupa [i,2],1)*equals(mobokupa[i,3],0)*equals(mobokupa [i,4],1)*equals (mobokupa [i,6],1)*q[i,5]

+ equals(mobokupa [i,2],1)*equals(mobokupa[i,3],0)*equals(mobokupa [i,4],1)*equals (mobokupa [i,6],0)*q[i,6]

+ equals(mobokupa [i,2],1)*equals(mobokupa[i,3],0)*equals(mobokupa [i,4],0)*equals (mobokupa [i,6],1)*q[i,7]

+ equals(mobokupa [i,2],1)*equals(mobokupa[i,3],0)*equals(mobokupa [i,4],0)*equals (mobokupa [i,6],0)*q[i,8]

+ equals(mobokupa [i,2],0)*equals(mobokupa[i,3],1)*equals(mobokupa [i,4],1)*equals (mobokupa [i,6],1)*q[i,9]

+ equals(mobokupa [i,2],0)*equals(mobokupa[i,3],1)*equals(mobokupa [i,4],1)*equals (mobokupa [i,6],0)*q[i,10]

+ equals(mobokupa [i,2],0)*equals(mobokupa[i,3],1)*equals(mobokupa [i,4],0)*equals (mobokupa [i,6],1)*q[i,11]

+ equals(mobokupa [i,2],0)*equals(mobokupa[i,3],1)*equals(mobokupa [i,4],0)*equals (mobokupa [i,6],0)*q[i,12]

+ equals(mobokupa [i,2],0)*equals(mobokupa[i,3],0)*equals(mobokupa [i,4],1)*equals (mobokupa [i,6],1)*q[i,13]

+ equals(mobokupa [i,2],0)*equals(mobokupa[i,3],0)*equals(mobokupa [i,4],1)*equals (mobokupa [i,6],0)*q[i,14]

+ equals(mobokupa [i,2],0)*equals(mobokupa[i,3],0)*equals(mobokupa [i,4],0)*equals (mobokupa [i,6],1)*q[i,15]

+ equals(mobokupa [i,2],0)*equals(mobokupa[i,3],0)*equals(mobokupa [i,4],0)*equals (mobokupa [i,6],0)*q[i,16]

) +(1-equals(valid[i],1)) *(1e-14);

#######################################################

## Ensure the probabilities are always less than 1

#######################################################

##Since in a Bernoulli density an observation of 1 has a likelihood of p[i]

p[i] <- L[i] / 1;## divided by a constant just to ensure all p's <1

ones[i] ~ dbern(p[i]);

}

#######################################################

## Definition of model priors

#########################################################

prc~dbeta(1,1);

c1~dbeta(1,1); # Specificity SICT test

c2~dbeta(1,1); # Specificity Bovigam® assay

c3<-1; # Specificity culture fixed

c4~dbeta(1,1); # Specificity necropsy

s1~dbeta(1,1); # Sensitivity SICT test

s2~dbeta(1,1); # Sensitivity Bovigam® assay

s3~dbeta(1,1); # Sensitivity culture

s4~dbeta(1,1); # Sensitivity necropsy

## Covariance terms

cs12<-0;

cs13<-0;

cs23<-0;*#~dbeta(1,1);*

cs14<-0;

cs24<-0;

cs34<-0;

cc12<-0;

cc13<-0;

cc23<-0;

cc14<-0;

cc24<-0;

cc34<-0;

logL<-sum(log(p[1:N]));

}
