# Supplementary material for: Evaluating diagnostic tests for bovine tuberculosis in the southern part of Germany: A latent class analysis
Source: PLoS One. 2017 Jun 22;12(6):e0179847. doi: 10.1371/journal.pone.0179847 (PMC5481003; doi:10.1371/journal.pone.0179847)
Supplement: S1 Table — b cut-off: 0.1. (DOCX) [file pone.0179847.s002.docx]

**S1 Table: Number of test result combinations in the four-test dataset (n=175), the inconclusive test results of the SICT test once considered as negative (standard interpretation) and once as positive (severe interpretation)**

| SICT test | Bovigam® assay^b^ | Culture | Necropsy | No of animals  SICT test as standard interpretation | No of animals  SICT test as severe interpretation |
| --- | --- | --- | --- | --- | --- |
|  |  |  |  |  |  |
| + | + | + | + | 8 | 9 |
| + | + | + | - | 0 | 2 |
| + | + | - | + | 0 | 1 |
| + | + | - | - | 38 | 149 |
| + | - | + | + | 1 | 1 |
| + | - | + | - | 0 | 0 |
| + | - | - | + | 0 | 0 |
| + | - | - | - | 1 | 8 |
| - | + | + | + | 1 | 0 |
| - | + | + | - | 2 | 0 |
| - | + | - | + | 1 | 0 |
| - | + | - | - | 115 | 4 |
| - | - | + | + | 0 | 0 |
| - | - | + | - | 0 | 0 |
| - | - | - | + | 0 | 0 |
| - | - | - | - | 8 | 1 |
| Total | | | | 175 | 175 |

^b^ cut-off: 0.1
