# Supplementary material for: Evaluating diagnostic tests for bovine tuberculosis in the southern part of Germany: A latent class analysis
Source: PLoS One. 2017 Jun 22;12(6):e0179847. doi: 10.1371/journal.pone.0179847 (PMC5481003; doi:10.1371/journal.pone.0179847)
Supplement: S2 Table — (DOCX) [file pone.0179847.s003.docx]

**S2 Table: Number of test result combinations in the three-test dataset (n=389), the inconclusive test results of the SICCT test once considered as negative (standard interpretation) and once as positive (severe interpretation)**

| SICCT test | PCR | Necropsy | No of animals  [SICCT test as standard interpretation] | No of animals  [SICCT test as severe interpretation] |
| --- | --- | --- | --- | --- |
| + | + | + | 26 | 47 |
| + | + | - | 3 | 6 |
| + | - | + | 8 | 13 |
| + | - | - | 26 | 282 |
| - | + | + | 24 | 3 |
| - | + | - | 3 | 0 |
| - | - | + | 5 | 0 |
| - | - | - | 294 | 38 |
| Total | | | 389 | 389 |
