# Supplementary material for: Evaluating diagnostic tests for bovine tuberculosis in the southern part of Germany: A latent class analysis
Source: PLoS One. 2017 Jun 22;12(6):e0179847. doi: 10.1371/journal.pone.0179847 (PMC5481003; doi:10.1371/journal.pone.0179847)
Supplement: S3 Table — Model 1: no covariances; Model 2: covariance sensitivity SICT test, Bovigam® assay; Model 3: covariance sensitivity SICT test, culture; Model 4: covariance sensitivity SICT test, pathology; Model 5: covariance sensitivity Bovigam® assay, culture; Model 6: covariance sensitivity Bovigam® assay, pathology; Model 7: covariance sensitivity culture, pathology; CI, credibility interval; se, sensitivity; sp, specificity. (DOCX) [file pone.0179847.s004.docx]

**S3 Table: DIC, prevalence and diagnostic test accuracies of different models, without and with covariances of the sensitivities between the different tests, considered from the dataset (n=175) tested with SICT test [standard interpretation, uninformative priors], Bovigam® assay [cut-off=0.1], culture [sp=100%] and necropsy**

| Model | DIC | Prevalence (95% CI) | SICT test (95% CI) | | Bovigam® assay (95% CI) | | Culture (95% CI) | | Necropsy (95% CI) | |
| --- | --- | --- | --- | --- | --- | --- | --- | --- | --- | --- |
|  |  |  | se | sp | se | sp | se | sp | se | sp |
| 1 | 393.7 | 7.7 (4.2-12.3) | 70.3 (44.9-90.5) | 75.8 (68.8-82.2) | 95.7 (91.3-99.2) | 6.9 (3.6-11.1) | 88.9 (65.5-99.7) | fixed at 100 | 76.8 (51.6-94.4) | 99.0 (96.8-100) |
| 2 | 395.0 | 8,0 (4,3-12,8) | 71,3 (47,2-90,3) | 75.9 (69.2-82.2) | 94.8 (90.4-98.3) | 7.4 (4.0-11.8) | 81.1(56.1-97.2) | fixed at 100 | 70.9 (46.9-90.3) | 99.0 (96.9-100) |
| 3 | 394.6 | 8.1 (4.4-13.1) | 70.1 (46.7-89.1) | 75.9 (69.0-82.2) | 94.7 (90.2-98.4) | 7.5 (4.0-11.8) | 81.3 (57.6-97.1) | fixed at 100 | 70.1 (46.1-90.1) | 99.0 (96.9-100) |
| 4 | 394.4 | 8.1 (4.3-12.9) | 69.0 (45.8-87.9) | 76.0 (69.3-82.2) | 94.7 (90-3-98.4) | 7.4 (4.1-11.7) | 79.9 (53.9-96.8) | fixed at 100 | 72.4 (48.6-90.9) | 99.0 (97.0-100) |
| 5 | 393.6 | 7.8 (4.2-12.4) | 66.5 (43.6-86.8) | 75.8 (69.1-82.1) | 94.9 (90.6-98.4) | 7.3 (4.0-11.5) | 87.4 (65.1-98.5) | fixed at 100 | 72.6 (48.6-91.4) | 99.0 (96.9-100) |
| 6 | 394.4 | 7.9 (4.3-12.6) | 65.6 (42.8-86.0) | 75.9 (67.0-82.2) | 94.8 (90.4-98.4) | 7.4 (4.0-11.7) | 81.8 (57.6-97.2) | fixed at 100 | 77.0 (52.6-93.9) | 99.0 (96.9-100) |
| 7 | 393.6 | 8.0 (4.3-12.9) | 65.0 (42.2-85.6) | 75.9 (69.0-82.3) | 94.7 (90.3-98.4) | 7.4 (4.1-11.8) | 82.3 (58.0-97.1) | fixed at 100 | 75.4 (51.0-92.7) | 99.0 (97.0-100) |

Model 1: no covariances

Model 2: covariance sensitivity SICT test, Bovigam® assay

Model 3: covariance sensitivity SICT test, culture

Model 4: covariance sensitivity SICT test, pathology

Model 5: covariance sensitivity Bovigam® assay, culture

Model 6: covariance sensitivity Bovigam® assay, pathology

Model 7: covariance sensitivity culture, pathology

CI, credibility interval

se, sensitivity

sp, specificity
