# Supplementary material for: Evaluating diagnostic tests for bovine tuberculosis in the southern part of Germany: A latent class analysis
Source: PLoS One. 2017 Jun 22;12(6):e0179847. doi: 10.1371/journal.pone.0179847 (PMC5481003; doi:10.1371/journal.pone.0179847)
Supplement: S4 Table — (DOCX) [file pone.0179847.s005.docx]

**S4 Table: Dichotomized test results of the Bovigam® assay for two different cut-offs**

|  | Positive | | Negative | | Total |
| --- | --- | --- | --- | --- | --- |
| Cut-off | n | % | n | % | n |
| >0.1 | 166 | 97.1 | 5 | 2.9 | 171 |
| >0.2 | 46 | 26.9 | 125 | 73.1 | 171 |
