# Supplementary material for: Evaluating diagnostic tests for bovine tuberculosis in the southern part of Germany: A latent class analysis
Source: PLoS One. 2017 Jun 22;12(6):e0179847. doi: 10.1371/journal.pone.0179847 (PMC5481003; doi:10.1371/journal.pone.0179847)
Supplement: S5 Table — PPV, positive predictive value; NPV, negative predictive value. (DOCX) [file pone.0179847.s006.docx]

**S5 Table: Positive and negative predictive values of the SICT test, Bovigam® assay, culture [sp=100%] and necropsy calculated from the prevalence and diagnostic test accuracies obtained from the models of Table 1.**

| Test | PPV % | NPV % | based on model |
| --- | --- | --- | --- |
| SICT test [standard interpretation] | 19.51 | 96.83 | 1 |
| SICT test [severe interpretation] | 8.07 | 96.37 | 4 |
| Bovigam® assay [cut-off = 0.1] | 7.90 | 95.06 | 1 |
| Bovigam® assay [cut-off = 0.2] | 8.54 | 94.25 | 3 |
| Culture | - | 99.08 | 1 |
| Necropsy | 86.50 | 98.08 | 1 |

PPV, positive predictive value; NPV, negative predictive value
