# Supplementary material for: Evaluating diagnostic tests for bovine tuberculosis in the southern part of Germany: A latent class analysis
Source: PLoS One. 2017 Jun 22;12(6):e0179847. doi: 10.1371/journal.pone.0179847 (PMC5481003; doi:10.1371/journal.pone.0179847)
Supplement: S6 Table — Model 1: no covariances; Model 2: covariance sensitivity SICCT test, PCR; Model 3: covariance sensitivity SICCT test, pathology; Model 4: covariance sensitivity PCR, pathology; Model 5: covariance specificity SICCT test, PCR; Model 6: covariance specificity SICCT test, pathology; Model 7: covariance specificity PCR, pathology; CI, credibility interval; se, sensitivity; sp, specificity. (DOCX) [file pone.0179847.s007.docx]

**S6 Table: DIC, prevalence and diagnostic test accuracies of different models, without and with covariances of the sensitivities and specificities between the different tests, considered from the dataset (n=389) tested with SICCT test [standard interpretation; prior information], PCR and necropsy**

| Models | DIC | Prevalence (95% CI) | SICCT test (95% CI) | | PCR (95% CI) | | Necropsy (95% CI) | |
| --- | --- | --- | --- | --- | --- | --- | --- | --- |
|  |  |  | se | sp | se | sp | se | sp |
| 1 | 756.4 | 17.2 (13.4-21.4) | 57.5 (46.5-68.1) | 91.5 (88.4-94.2) | 80.6 (69.1-90.6) | 99.1 (97.5-100) | 90.7 (80.7-98.0) | 99.1 (97.2-100) |
| 2 | 756.6 | 17.4 (13.6-21.5) | 57.3 (46.6-67.6) | 91.5 (88.4-94.1) | 79.1 (67.4-89.0) | 99.1 (97.6-100) | 89.5 (79.8-96.7) | 99.2 (97.4-100) |
| 3 | 756.3 | 17.5 (13.7-21.7) | 57,1 (46.3-67.6) | 91.5 (88.3-94.2) | 79.6 (68.3-89.3) | 99.3 (97.8-100) | 88.1 (77.9-96.1) | 99.1 (97.2-100) |
| 4 | 750.7 | 19.8 (14.6-26.5) | 57.8 (48.0-67.6) | 92.8 (89.2-96.3) | 70.6 (52.0-86.0) | 99.0 (97.4-99.9) | 78.4 (58.6- 93.7) | 98.9 (96.8-100) |
| 5 | 758.9 | 16.7 (12.9-20.8) | 58.1 (47.1-68.6) | 91.4 (88.3-94.1) | 82.1 (70.4-92.0) | 98.6 (96.7-99.7) | 92.0 (82.1-98.8) | 98.5 (96.4-99.7) |
| 6 | 758.9 | 16.7 (12.9-20.9) | 58.2 (47.3-69.1) | 91.4 (88.2-98.7) | 82.1 (70.6-92.1) | 98.6 (96.8-99.7) | 91.8 (82.2-98.7) | 98.5 (96.3-99.8) |
| 7 | 757.8 | 17.0 (13.1-21.2) | 58.0 (47.1-68.7) | 91.2 (88.0-94.0) | 81.2 (69.9-90.9) | 98.8 (97.1-99.8) | 91.1 (81.4-98.1) | 98.7 (96.9-99.8) |

Model 1: no covariances

Model 2: covariance sensitivity SICCT test, PCR

Model 3: covariance sensitivity SICCT test, pathology

Model 4: covariance sensitivity PCR, pathology

Model 5: covariance specificity SICCT test, PCR

Model 6: covariance specificity SICCT test, pathology

Model 7: covariance specificity PCR, pathology

CI, credibility interval

se, sensitivity

sp, specificity
