# Supplementary material for: Evaluating diagnostic tests for bovine tuberculosis in the southern part of Germany: A latent class analysis
Source: PLoS One. 2017 Jun 22;12(6):e0179847. doi: 10.1371/journal.pone.0179847 (PMC5481003; doi:10.1371/journal.pone.0179847)
Supplement: S7 Table — PPV, positive predictive value; NPV, negative predictive value. (DOCX) [file pone.0179847.s008.docx]

**S7 Table: Positive and negative predictive values of the SICCT test, PCR and necropsy calculated from the prevalence and diagnostic test accuracies obtained from the models of Table 3.**

| Test | PPV % | NPV % | based on model |
| --- | --- | --- | --- |
| SICCT Test [standard interpretation] | 66.46 | 89.91 | 3 |
| SICCT Test [severe interpretation] | 16.62 | 92.72 | 5 |
| PCR | 94.57 | 93.17 | 3 |
| Necropsy | 94.62 | 94.88 | 3 |

PPV, positive predictive value; NPV, negative predictive value
