# Supplementary material for: Evaluating diagnostic tests for bovine tuberculosis in the southern part of Germany: A latent class analysis
Source: PLoS One. 2017 Jun 22;12(6):e0179847. doi: 10.1371/journal.pone.0179847 (PMC5481003; doi:10.1371/journal.pone.0179847)
Supplement: S8 Table — Pos, positive; neg, negative; n.a., not analyzable. (DOCX) [file pone.0179847.s009.docx]

**S8 Table: Test results of the Bovigam® assay from five officially approved laboratories; the results of the time point with the best accordance of the proportion of positive test results is shown**

| Cow no | Laboratory 1 | Laboratory 2 | Laboratory 3 | Laboratory 4 | Laboratory 5 |
| --- | --- | --- | --- | --- | --- |
| 1 | pos | pos | pos | pos | neg |
| 2 | pos | neg | neg | pos | neg |
| 3 | pos | pos | pos | pos | pos |
| 4 | neg | neg | neg | neg | neg |
| 5 | pos | pos | pos | pos | neg |
| 6 | pos | pos | pos | pos | pos |
| 7 | pos | pos | pos | pos | neg |
| 8 | pos | pos | pos | pos | pos |
| 9 | neg | pos | pos | n.a. | neg |
| 10 | neg | pos | pos | n.a. | pos |
| 11 | pos | pos | pos | n.a. | neg |
| 12 | neg | pos | pos | pos | pos |
| 13 | pos | pos | pos | neg | pos |
| 14 | neg | pos | pos | neg | pos |
| 15 | pos | pos | pos | pos | pos |
| 16 | neg | neg | pos | pos | pos |
| 17 | pos | neg | pos | neg | neg |
| 18 | pos | pos | pos | pos | pos |
| 19 | pos | pos | pos | pos | pos |
| 20 | pos | pos | pos | pos | pos |
| 21 | neg | neg | neg | pos | neg |

Pos, positive; neg, negative; n.a., not analyzable
