# Supplementary material for: Evaluating diagnostic tests for bovine tuberculosis in the southern part of Germany: A latent class analysis
Source: PLoS One. 2017 Jun 22;12(6):e0179847. doi: 10.1371/journal.pone.0179847 (PMC5481003; doi:10.1371/journal.pone.0179847)
Supplement: S9 Table — The assayed blood was taken at two time points respectively from two differing localizations. V.j., V. jugularis; V.s.a., V. subcutanea abdominis; V.c.m., V. caudalis mediana; pos, positive; neg, negative;?, inconclusive; n.a., not analyzable. (DOCX) [file pone.0179847.s010.docx]

**S9 Table: Test results of the Bovigam® assay from five officially approved laboratories. The assayed blood was taken at two time points respectively from two differing localizations.**

| cow no | Laboratory 1 | | | | Laboratory 2 | | | | Laboratory 3 | | | | Laboratory 4 | | | | Laboratory 5 | | | |
| --- | --- | --- | --- | --- | --- | --- | --- | --- | --- | --- | --- | --- | --- | --- | --- | --- | --- | --- | --- | --- |
|  | *V.j. / V.s.a.* | | *V.j. / V.c.m.* | | *V.j. / V.s.a.* | | *V.j. / V.c.m.* | | *V.j. / V.s.a.* | | *V.j. / V.c.m.* | | *V.j. / V.s.a.* | | *V.j. / V.c.m.* | | *V.j. / V.s.a.* | | *V.j. / V.c.m.* | |
|  | *V.j.* | *V.s.a.* | *V.j.* | *V.c.m.* | *V.j.* | *V.s.a.* | *V.j.* | *V.c.m.* | *V.j.* | *V.s.a.* | *V.j.* | *V.c.m.* | *V.j.* | *V.s.a.* | *V.j.* | *V.c.m.* | *V.j.* | *V.s.a.* | *V.j.* | *V.c.m.* |
| 1 | neg | neg | pos | pos | pos | neg | pos | neg | pos | pos | pos | neg | pos | pos | pos | neg | pos | neg | neg | neg |
| 2 | neg | pos | pos | pos | pos | neg | neg | neg | neg | neg | neg | neg | neg | pos | pos | pos | pos | neg | neg | neg |
| 3 | pos | pos | pos | pos | pos | pos | pos | neg | pos | pos | pos | pos | pos | pos | pos | pos | pos | pos | pos | pos |
| 4 | neg | n.a. | neg | pos | neg | neg | neg | neg | n.a. | n.a. | n.a. | n.a. | neg | neg | neg | neg | neg | neg | neg | neg |
| 5 | pos | pos | pos | pos | pos | pos | pos | pos | pos | pos | pos | pos | pos | pos | pos | pos | pos | pos | neg | pos |
| 6 | pos | pos | pos | pos | pos | pos | pos | pos | pos | pos | pos | pos | neg | pos | pos | neg | pos | ? | pos | pos |
| 7 | pos | neg | pos | neg | pos | neg | pos | neg | pos | pos | pos | neg | pos | pos | pos | pos | neg | neg | neg | neg |
| 8 | pos | pos | pos | pos | pos | pos | pos | pos | pos | pos | pos | pos | pos | pos | pos | pos | pos | pos | pos | pos |
| 9 | neg | neg | neg | pos | n.a. | pos | pos | pos | pos | pos | pos | pos | pos | neg | n.a. | neg | neg | neg | neg | pos |
| 10 | pos | pos | neg | pos | pos | pos | pos | pos | pos | pos | pos | pos | neg | neg | n.a. | neg | pos | pos | pos | pos |
| 11 | pos | pos | pos | pos | pos | pos | pos | pos | pos | pos | pos | pos | neg | neg | n.a. | neg | pos | ? | neg | pos |
| 12 | pos | neg | neg | neg | pos | pos | pos | pos | pos | pos | pos | pos | neg | neg | pos | pos | pos | neg | pos | pos |
| 13 | pos | pos | pos | pos | pos | pos | pos | pos | pos | pos | pos | pos | pos | pos | neg | pos | pos | pos | pos | pos |
| 14 | pos | pos | neg | pos | pos | pos | pos | pos | pos | pos | pos | pos | pos | pos | neg | pos | pos | pos | pos | pos |
| 15 | pos | pos | pos | pos | pos | pos | pos | pos | pos | pos | pos | pos | pos | pos | pos | pos | pos | pos | pos | pos |
| 16 | neg | pos | neg | pos | pos | pos | neg | pos | pos | pos | pos | pos | pos | pos | pos | neg | pos | neg | pos | pos |
| 17 | pos | pos | pos | pos | pos | neg | neg | neg | pos | pos | pos | pos | neg | pos | neg | pos | pos | neg | neg | neg |
| 18 | pos | pos | pos | pos | pos | pos | pos | pos | pos | pos | pos | pos | pos | pos | pos | pos | pos | pos | pos | pos |
| 19 | pos | pos | pos | pos | pos | pos | pos | pos | pos | pos | pos | pos | pos | pos | pos | pos | pos | ? | pos | pos |
| 20 | pos | pos | pos | neg | pos | pos | pos | pos | pos | pos | pos | pos | pos | pos | pos | pos | pos | neg | pos | pos |
| 21 | neg | pos | neg | neg | neg | neg | neg | neg | neg | neg | neg | neg | neg | pos | pos | neg | neg | neg | neg | neg |

*V.j., V. jugularis; V.s.a., V. subcutanea abdominis; V.c.m., V. caudalis mediana*; pos, positive; neg, negative; ?, inconclusive; n.a., not analyzable
