# Supplementary material for: Evaluating diagnostic tests for bovine tuberculosis in the southern part of Germany: A latent class analysis
Source: PLoS One. 2017 Jun 22;12(6):e0179847. doi: 10.1371/journal.pone.0179847 (PMC5481003; doi:10.1371/journal.pone.0179847)

**S1 Fig: Histograms from the covariances of the sensitivities between the different tests, considered from the four-test dataset (n=175) tested with SICT test [standard interpretation; no prior information], Bovigam® assay [cut−off=0.1], culture [sp=100%] and necropsy**

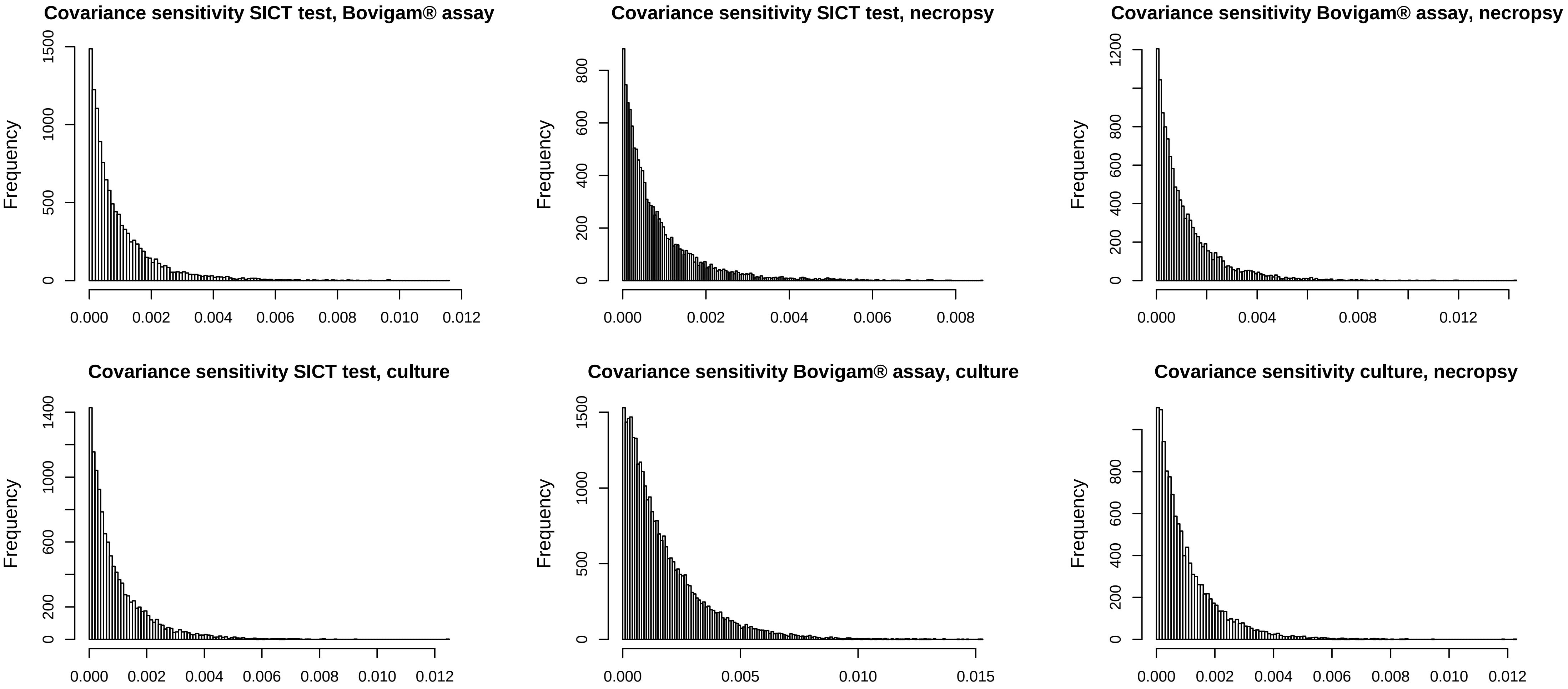

Supplement: S1 Fig — (PDF) [file pone.0179847.s011.pdf]
