# Supplementary material for: Evaluating diagnostic tests for bovine tuberculosis in the southern part of Germany: A latent class analysis
Source: PLoS One. 2017 Jun 22;12(6):e0179847. doi: 10.1371/journal.pone.0179847 (PMC5481003; doi:10.1371/journal.pone.0179847)

S2 Fig: Histograms from the covariances of the sensitivities and specificities between the different tests, considered from the three-test dataset (n=389) tested with SICCT test [standard interpretation; prior information], PCR and necropsy

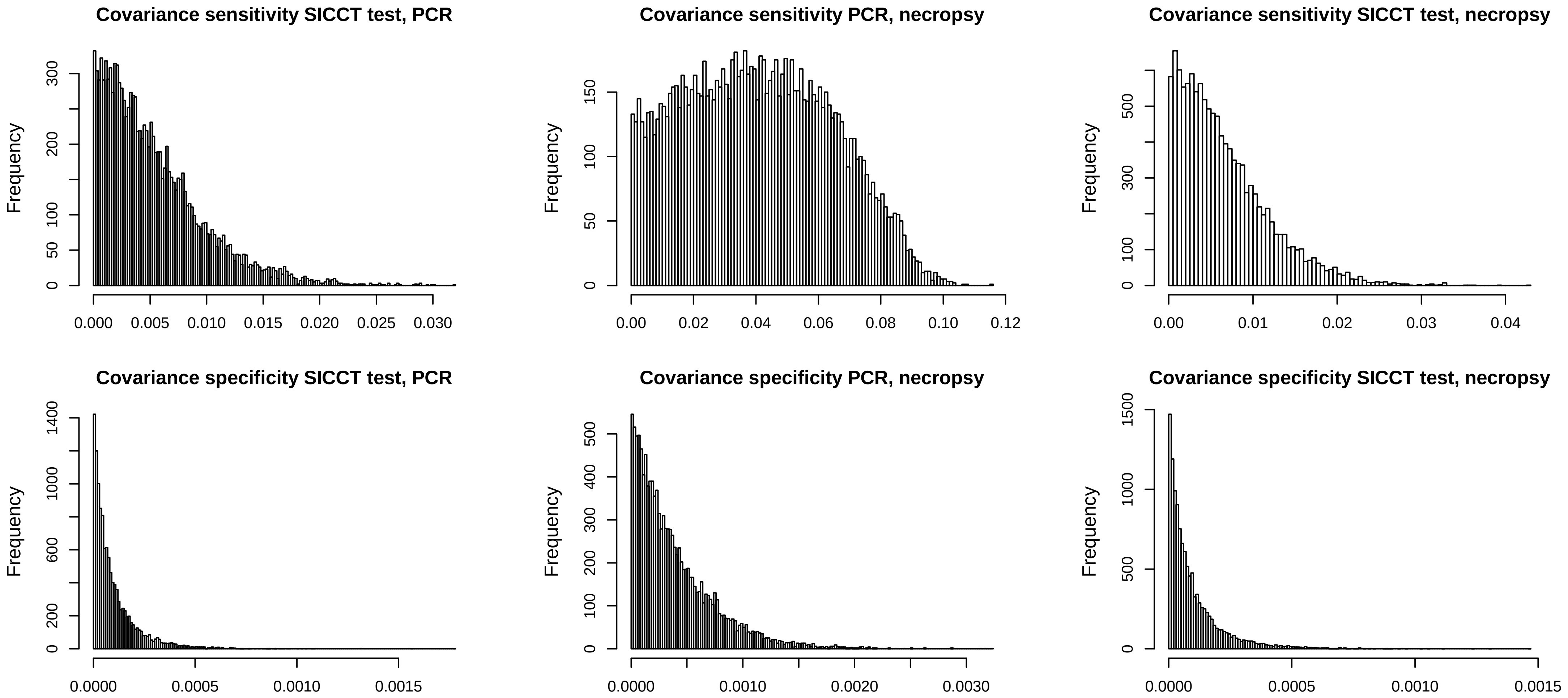

Supplement: S2 Fig — (PDF) [file pone.0179847.s012.pdf]
